# Vascular Endothelial Growth Factor (VEGF) Family and the Immune System: Activators or Inhibitors?

**DOI:** 10.3390/biomedicines13010006

**Published:** 2024-12-24

**Authors:** Cristina Maria Failla, Maria Luigia Carbone, Carmela Ramondino, Emanuele Bruni, Angela Orecchia

**Affiliations:** 1 Experimental Immunology Laboratory, Istituto Dermopatico dell’Immacolata, IDI-IRCCS, 00167 Rome, Italy; c.failla@idi.it (C.M.F.); c.ramondino@idi.it (C.R.); 2 Clinical Trial Center, Istituto Dermopatico dell’Immacolata, IDI-IRCCS, 00167 Rome, Italy; marialuigia.carbone@idi.it; 3 Departmental Faculty of Medicine and Surgery, UniCamillus-Saint Camillus International University of Health Sciences, 00131 Rome, Italy; 4 ContendEd Net, 00166 Rome, Italy; angela.orecchia@contentednet.com

**Keywords:** vascular angiogenic growth factor, immune cells, tyrosine kinase inhibitor, immunotherapy, angiogenesis

## Abstract

The vascular endothelial growth factor (VEGF) family includes key mediators of vasculogenesis and angiogenesis. VEGFs are secreted by various cells of epithelial and mesenchymal origin and by some immune cells in response to physiological and pathological stimuli. In addition, immune cells express VEGF receptors and/or co-receptors and can respond to VEGFs in an autocrine or paracrine manner. This immunological role of VEGFs has opened the possibility of using the VEGF inhibitors already developed to inhibit tumor angiogenesis also in combination approaches with different immunotherapies to enhance the action of effector T lymphocytes against tumor cells. This review pursues to examine the current understanding of the interplay between VEGFs and the immune system, while identifying key areas that require further evaluation.

## 1. Introduction

Blood vessel formation and organization occur through two processes defined as vasculogenesis and angiogenesis. Vasculogenesis is the development of a capillary network due to differentiation of pluripotent mesenchymal cells into hemangioblasts, while angiogenesis refers to the process by which new blood vessels develop from existing ones [1]. The importance of vasculogenesis and angiogenesis is linked to their homeostatic role of being suppliers of oxygen and nutrients to tissues and organs, and of removing, at the same time, discarded metabolites. Therefore, vasculogenesis and angiogenesis represent crucial points of physiological processes such as embryonic development, growth, hematopoiesis, tissue remodeling, and wound healing, but also of pathological conditions, such as cancer, inflammation, atherosclerosis, or diabetic retinopathy [1,2]. These processes are coordinated both by the interplay between different cell types of endothelial and non-endothelial origin, and by cell responses to angiogenic or anti-angiogenic factors [3].

The vascular endothelial growth factor (VEGF) family comprises central mediators in the vasculogenesis and angiogenesis processes as highlighted by studies with knockout mice [4]. Since 1983, with the isolation of the vascular permeability factor VPF/VEGF-A [5], the most-studied member of the family, the role of the diverse VEGFs and their receptors has been deeply characterized either in physiological or pathological angiogenesis [6].

Due to the possibility of blocking tumor angiogenesis by acting on VEGF-A, pharmacological studies have led to the approval of a monoclonal antibody (mAb) against VEGF-A (bevacizumab/Avastin) in cancer therapy. Next, tyrosine kinase inhibitors targeting VEGFRs, such as sorafenib and sunitinib, have gained approval for the treatment of various cancers, either as standalone therapies or in combination with chemotherapy or radiotherapy [7]. Anti-VEGF-A mAbs, specifically formulated for use in the eye (ranibizumab, aflibercept, and brolucizumab), have been also approved for the treatment of maculopathies [8].

Following his intuition on tumor angiogenesis, Judah Folkman was also the pioneer who suggested that immune cells may influence angiogenesis and vice versa [9]. In fact, immune cells produce several growth factors and cytokines that modulate both angiogenesis and lymphangiogenesis [10]. In addition, the role of angiogenic growth factors and, in particular, of the VEGF family members in the modulation of anti-tumor immunity, has been investigated [11,12]. Indeed, the role of VEGF-A in contributing to the tumor immunosuppressive microenvironment has been highlighted [13].

Application of immunotherapy in diverse types of cancer, such as renal cell carcinoma, previously treated with angiogenic inhibitors targeting the VEGF family, has led to the proposal of combined therapeutic protocols [14,15]. Therefore, understanding the functional connections among VEGFs, VEGF receptors (VEGFR) and co-receptors, and the immune system has become more and more important.

This review intends to recapitulate available knowledge in this field and underline the aspects that still need deeper investigation.

## 2. VEGF Family Members and Immune Cells

The mammalian VEGF family is composed of five growth factors, VEGF-A, VEGF-B, placenta growth factor (PlGF), VEGF-C, and VEGF-D, that recognize, with different affinities, the VEGFR cellular receptors (VEGFR-1, VEGFR-2, and VEGFR-3) [16]. Membrane VEGFRs present three different domains: an extracellular domain that binds to the growth factor, a transmembrane domain, and an intracellular domain with tyrosine kinase activity. VEGF-A interacts with both VEGFR-1 and VEGFR-2, while VEGF-B and PlGF are specific to VEGFR-1, and VEGF-C along with VEGF-D bind exclusively to VEGFR-3. Selected isoforms of these growth factors, generated through alternative splicing of the same gene, specifically recognize and bind to the non-tyrosine kinase receptors neuropilin-1 (NRP-1) and neuropilin-2 (NRP-2). Other molecules can interact with the VEGFs, such as integrins, cadherins, or heparin sulfate proteoglycans, but their specific role and effects have been less characterized.

### 2.1. VEGF-A

VEGF-A was first characterized as a permeability factor and then for its actions on endothelial cell proliferation, vasodilation, and inhibition of endothelial cell apoptosis [17]. It plays a crucial role in the maintenance of physiological vascular homeostasis in different tissues, and its involvement in the pathogenesis of diseases such as tumor growth and metastases and diabetic and hypertensive retinopathy has been demonstrated [5].

The different VEGF-A structures derived from alternative splicing determine protein–protein interactions that can modulate growth factor functions. Exons 6 and 7, which are lacking in some isoforms, are responsible for binding to heparin and extracellular matrix heparin sulfate proteoglycans, and exon 7 for VEGF-A interaction with NRP-1 and NRP-2 [18].

**Figure 1:**
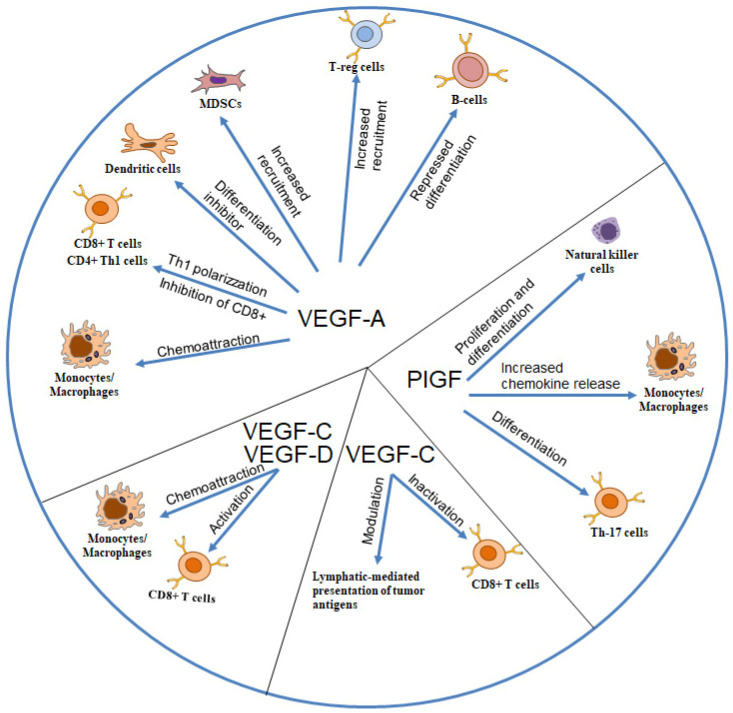
Schematic representation of the different interactions among VEGFs and immune cells. VEGF-A has been reported to interact with monocytes/macrophages, dendritic cells, mesenchymal-derived suppressor cells (MDSCs), and regulatory T cells (T-reg) by acting as a chemoattractant factor [17,19,22]. VEGF-A increases polarization of T cells towards the T helper (h) 1 phenotype [21] and inhibits differentiation of dendritic cells and B cells [20]. PlGF increases chemokine release by monocytes/macrophages and induces differentiation of Natural killer cells and Th-17 lymphocytes [23,24,25]. VEGF-C and, probably, also VEGF-D, act as chemoattractants for monocytes/macrophages and induce activation of CD8+ T cells [26]. Conversely, VEGF-C can modulate lymphatic-mediated presentation of tumor antigens and inactivate CD8+ T cells [27]. Parts of the figure are drawn using pictures from Servier Medical Art (https://smart.servier.com).

VEGF-A mainly interacts with immune cells by promoting a chemotactic response that recruits dendritic cells (DCs), myeloid-derived suppressor cells (MDPCs), and macrophages/monocytes to the damaged tissue where they contribute to maintaining local inflammatory processes by increasing the release of proinflammatory cytokines (Figure 1) [17,19].

VEGF-A is also involved in the recruitment of precursor DCs and in their trans-differentiation within the tissue into endothelial-like cells (Figure 1) [20]. VEGF-A increases interferon (IFN)-γ production, suppresses interleukin (IL)-10 secretion, and promotes the polarization of T cells toward a T helper (Th)1 phenotype (Figure 1) [21].

### 2.2. VEGF-B and PlGF

VEGF-B is expressed in early embryonic stages and contributes to the development of the cardiovascular system and myocardium, but its role in vasculogenesis is not essential, as demonstrated by the VEGF-B knockout mice, which are viable at birth and exhibit moderate cardiovascular defects. Two VEGF-B isoforms result from alternative splicing: VEGF-B167, which binds to ECM and interacts with VEGFR-1 and NRP-1, and VEGF-B186, which interacts only with VEGFR-1 and gains the ability to bind NRP-1 upon cleavage [28]. In adults, VEGF-B is found in various tissues such as myocardium, pancreas, and skeletal muscle, but its role as an angiogenic factor is still controversial. In fact, VEGF-B is mainly associated with the survival of smooth muscle cells, pericytes, neurons, and cardiomyocytes [29]. VEGF-B is upregulated in diverse types of cancer [29]. However, in the RIP1-Tag2 mouse model of pancreatic neuroendocrine tumorigenesis, VEGF-B over-expression reduced tumor growth [30]. No differences in vascular density, perfusion, or immune cell infiltration were seen in these experiments [30].

To date, no data have been reported that specifically highlight the role of VEGF-B in immune cells. VEGF-B is overexpressed by macrophages in certain inflammatory conditions such as obesity and is involved in the development of obesity-associated hypertension [31].

PlGF was originally identified in the human placental tissue and its expression correlated with trophoblast growth and differentiation, trophoblast invasion, and blastocyst implantation. In adults, PlGF is found in four isoforms (PlGF-1, PlGF-2, PlGF-3, and PlGF-4) with different structure and activity. All isoforms bind and activate VEGFR-1; the PlGF-2 isoform also interacts with NRP-1, NRP-2, and heparin.

PlGF significantly induces expression of inflammatory chemokines, such as tumor necrosis factor (TNF)-α, IL-1β, monocyte chemotactic protein (MCP)-1, IL-8, and VEGF-A, by monocytes, contributing to sustaining monocyte activation (Figure 1) [32,33]. PlGF specifically induces the production of transforming growth factor (TGF)-β1 by tumor-associated macrophages (Figure 1) [23].

Analyzing the implantation site in PlGF null mice, Tayade et al. found a role for PlGF in uterine natural killer (NK) cell proliferation and differentiation (Figure 1) [24]. Uterine NK cells are primarily responsible for early vascular changes in the pregnant endometrium. They also express VEGF-A. However, several studies have failed to demonstrate the presence of VEGFR-1 on uterine NK cells, and the mechanism of PlGF- or VEGF-A-dependent activation of uterine NK cells is still unclear. T cells express VEGFR-1 and PlGF transduces its differentiation program through activation of this tyrosine kinase receptor. The analysis of splenocytes from PlGF-null mice revealed significantly reduced levels of IL-17 compared to wild-type control. Further investigations demonstrated that PlGF specifically regulates the differentiation of Th cells towards the Th17 lineage (Figure 1) [25].

### 2.3. VEGF-C and VEGF-D

VEGF-C is expressed in the embryonic tissues, where it regulates lymphangiogenesis by binding and activating VEGFR-3. Upon processing, VEGF-C is also able to bind to VEGFR-2. VEGF-C activity can be increased by binding to NRP-2, which acts as a co-receptor for VEGFR-3 [34]. VEGF-C inactivation impairs lymphatic vessel development, resulting in lethal accumulation of interstitial fluids in the tissues. In adult life, VEGF-C is involved in lymphangiogenesis in both physiological and pathological situations [35].

VEGF-D is highly expressed in the embryonic lungs, where it also participates in the development of the lymphatic vessels. Like VEGF-C, VEGF-D binds to VEGFR-3 and NRP-2. However, VEGF-D inactivation is not lethal, resulting in a moderate atrophy of lymphatic circulation [36].

In the tumor microenvironment, the presence of VEGF-C or VEGF-D could be linked to migration of tumor cells into lymphatic vessels and the formation of lymph node metastasis [37].

Neutrophils contribute to lymphangiogenesis by secretion of VEGF-C [38]. Steroids and TNF-α strongly induce VEGF-C in MSCs, which subsequently enhances CD8+ T cell responses. This immune-promoting effect is completely prevented by blocking VEGFR3 or by specifically deleting VEGFR3 in CD8+ T cells (Figure 1) [26]. In a mouse model of melanoma, Lund et al. showed that VEGF-C-activated lymphatic endothelial cells take up and cross-present tumor antigens, leading to dysfunctional activation of CD8+ T cells [27].

## 3. Regulation of VEGF-A Expression in Immune Cells

**Figure 2:**
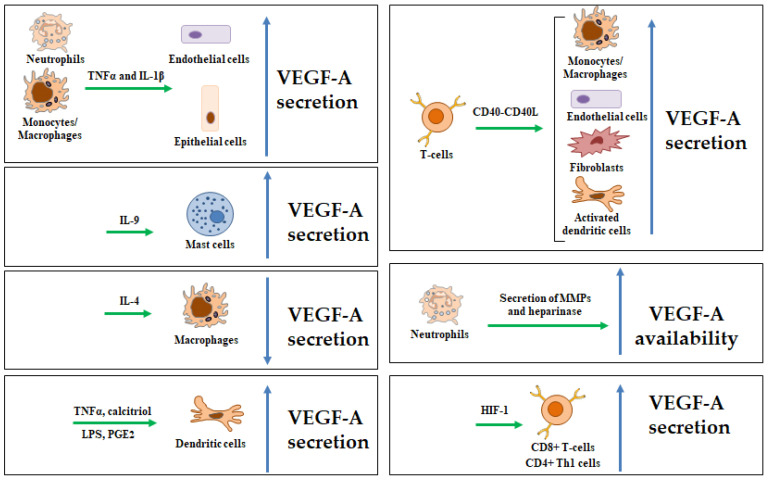
Immune cell-mediated mechanisms of regulation of VEGF-A expression. Neutrophils and monocytes/macrophages release inflammatory cytokines (TNFα and IL-1β), which stimulate the secretion of VEGF-A by both endothelial and non-endothelial cells [40]. IL-9 induces VEGF-A release by human mast cells [41], whereas IL-4 inhibits the release of VEGF-A by human macrophages [42]. CD40-CD40L-mediated cell–cell contact can induce VEGF-A secretion by different cell types [43,44]. VEGF-A secretion by dendritic cells is induced by molecules such as TNFα, calcitriol, lipopolysaccharide (LPS), or prostaglandin (PG)E_2_. Neutrophils augment VEGF-A availability by secretion of matrix metalloproteinases (MMPs) and heparinases [38], and hypoxia can induce T cells to secrete VEGF-A through hypoxia-inducible factor (HIF)-1 [21,45]. Parts of the figure are drawn using pictures from Servier Medical Art (https://smart.servier.com).

VEGF-A is secreted by different cell types of epithelial and mesenchymal origin, and its expression is mainly modulated by hypoxia [39]. Several immune cell growth factors and inflammatory mediators can also induce VEGF-A expression, including platelet-derived growth factor-BB, epidermal growth factor (EGF), TGF-β1, IL-1β, and TNF-α (Figure 2) [40]. IL-9 is a cytokine with pleiotropic functions in immune cells. IL-9 induces VEGF-A secretion by human mast cells, which contributes to the progression of atopic dermatitis, an inflammatory skin disease [41]. Conversely, IL-4 can simultaneously inhibit the secretion of VEGF-A and augment the secretion of the anti-angiogenic soluble form of VEGFR-1 in human macrophages [42].

In the presence of proinflammatory molecules, such as lipopolysaccharide (LPS), TNF-α, and IL-1β, classically activated DCs express VEGF-A together with high levels of anti-angiogenic molecules. Conversely, alternatively activated DCs mature in the presence of anti-inflammatory molecules, such as calcitriol or prostaglandin E2 (PGE2), and produce elevated levels of VEGF-A. This promotes vascular growth at sites of tissue inflammation or wounds, as well as in reactive lymph nodes (Figure 2) [20]. These findings indicate that the presence of pro- or anti-inflammatory mediators in the tissue microenvironment is the principal responsible for production of VEGF-A by DCs.

Immune cell contact-mediated activation can lead to VEGF-A induction. CD40L expressed on T cells engages CD40 in endothelial cells, monocytes, and fibroblast-like synoviocytes, resulting in strong VEGF-A induction (Figure 2) [43,44].

VEGF-A is secreted by T cells upon stimulation by IL-2 or hypoxia through the hypoxia-inducible factor (HIF)-1 [21,45]. Hypoxia also triggers the expression of VEGFR-2 in T cells, enhancing the responses of these cells to VEGF-A. Neutrophils can modulate VEGF-A bioavailability and bioactivity via secretion of matrix metalloproteinases and heparinase (Figure 2) [38].

Epigenetic factors can also regulate VEGF-A expression in tumor cells [40]. MicroRNA as well as long non-coding RNAs or circular RNAs are emerging as crucial modulators of VEGF-A expression in cancer [46,47]. However, this kind of regulation was not specifically analyzed in immune cells.

## 4. Receptors of the VEGF Family and Their Expression on Immune Cells

### 4.1. VEGFR-1

VEGFR-1, formerly referred to as the Fms-like tyrosine kinase (Flt)-1 receptor, plays a critical role in the development and maturation of the embryonic vasculature. Notably, embryos lacking VEGFR-1 die in utero between days 8.5 and 9.0 [48]. The defect was later attributed to an excessive proliferation of endothelial cells and premature commitment of angioblasts, which disrupted the proper organization of the vascular network. This was likely due to the absence of the inhibitory effect of VEGFR-1 on VEGF-A [49]. In fact, VEGF-A reacts with higher affinity with VEGFR-1, without inducing a clear tyrosine kinase activation of the receptor but impeding VEGF-A from properly interacting with its functional VEGFR-2 [50]. In addition to the membrane-bound form, endothelial cells also produce a soluble isoform of VEGFR-1 (sVEGFR-1). This isoform binds to members of the VEGF family, reducing the availability of VEGFs for interaction with their membrane receptors, thereby inhibiting VEGFR-mediated signaling. Furthermore, sVEGFR-1 plays a supplementary role in angiogenesis by being deposited in the extracellular matrix, where it interacts with components of the cell membrane [51]. The binding of sVEGFR-1 to α5β1 integrin on endothelial cells regulates vessel growth, inducing a dynamic, pro-angiogenic phenotype [52]. Therefore, the amount of and the location of sVEGFR-1 in the tissue microenvironment regulate the activation or inhibition of cells expressing membrane VEGFR-1 (mVEGFR-1) or α5β1 integrin.

Different immune cells express mVEGFR-1 and react to VEGFR-1-binding growth factors such as VEGF-A, VEGF-B, and PlGF.

Human DC precursors express mVEGFR-1 and DC maturation is inhibited by a VEGFR-1-dependent signaling triggered by either VEGF-A or PlGF [20]. No data about a similar action of VEGF-B have been reported so far.

Acute lung injury is characterized by hyperinflammation and involves reduced levels of alveolar macrophage and recruitment of monocyte-derived macrophages in a VEGFR-1-dependent manner. The monocyte-derived macrophages that express VEGFR-1 displayed an anti-inflammatory phenotype [53]. Conversely, VEGFR-1 expression is up-regulated in tumor-associated macrophages and VEGFR-1 activation sustains polarization toward the M2 phenotype [54].

Bone marrow-derived hematopoietic progenitor cells, mobilized by VEGFs and other tumor-associated factors, also express VEGFR-1, in addition to monocytes/macrophages [55]. These cells are able to migrate to distant sites before the arrival of metastatic tumor cells and are essential in the formation and maintenance of pre-metastatic niches [56].

So far, data on sVEGFR-1 secretion are available only for human macrophages [42]. Different immune cells express the α5β1 integrin [57] and could respond to the stimulation of sVEGFR-1 in the extracellular matrix. This aspect still requires additional investigation.

### 4.2. VEGFR-2

VEGFR-2, previously known as the kinase insert domain receptor (KDR), is predominantly expressed on endothelial cells of both blood and lymphatic vessels.

The importance of VEGFR-2 was revealed by knockout experiments in which the inactivation of VEGFR-2 in mice resulted in a lack of vasculogenesis, leading to embryonic death. In adults, VEGFR-2 regulates angiogenesis, through VEGF-A-mediated signal transduction, and lymphangiogenesis, by interacting with VEGF-C and VEGF-D [58].

Monocyte-derived mature DCs express both VEGFR-1 and VEGFR-2. However, VEGF-A suppresses the antigen-presenting function of mature DCs by interacting specifically with VEGFR-2 [59,60]. Similarly, MDSCs are recruited by VEGF-A in a VEGFR-2-dependent manner [22].

So far, VEGF-A is considered the major protein responsible for the immunosuppressive environment in tumors [61], even if the expression of VEGFR-2 on T cells is controversial. In vitro-activated T cells as well as tumor-infiltrating T cells express VEGFR-2 [62]. The VEGF-A/VEGFR-2 axis is involved in the induction of either T cell proliferation or tumor-induced T cell exhaustion, the latter through increased expression of immune checkpoint molecules [63,64].

VEGF-A induces proliferation of Tregs through binding and activating VEGFR-2, but only certain Treg subclasses express VEGFR-2 [22]. It would be of interest to evaluate the prognostic role of tumor-infiltrating VEGFR-2-positive Tregs. This selective evaluation could be more accurate for patient prognosis than considering all Tregs.

### 4.3. VEGFR-3

VEGFR-3, or Fms-like tyrosine kinase (Flt)-4, plays a crucial role both in the formation of the lymphatic vessel network during embryonic development and in the formation of adult lymphatic vessels. A soluble sVEGFR-3 was also found, which inhibited lymphatic vessel development by interfering with the signals triggered by VEGF-C and VEGF-D [65].

In the immune system, VEGFR-3 is expressed by macrophages and promotes cell chemotaxis and activation [26,66].

VEGFR-3 signaling plays a role in modulating allergic airway inflammation. During acute inflammation, blocking VEGFR-3 reduces immune cell recruitment to the lungs. However, in the absence of VEGFR-3, the memory response to allergens is significantly worsened. This response relies on the lack of both VEGF-C and VEGF-D, suggesting that these two growth factors might compensate for one another in allergic airway inflammation [67].

### 4.4. NRP-1 and NRP-2

NRP-1 and NRP-2 were initially identified as receptors for semaphorins (SEMA) involved in neuron system development [68]. NRP-1 promotes the interaction of SEMA3 with plexins, mediating axon guidance during embryonic development. Subsequently, additional growth factors have been shown to bind NRP-1 and NRP-2, such as TGF-β, hepatocyte growth factor, or platelet-derived growth factor, as well as integrins and small molecules such as synectin [69], but the respective roles in angiogenesis have not been fully characterized yet.

NRPs are non-tyrosine kinase transmembrane receptors and act as co-receptors for different isoforms of VEGFs [70]. Both NRP-1 and NRP-2 exist as either membrane-bound or soluble forms that display decoy functions to membrane NRPs [71]. Endothelial cells of arteries express primarily NRP-1, which possess a selective binding site for VEGF-A165, PlGF-2, and VEGF-B167. The co-expression of NRP-1 and VEGFR-2 enhances the binding of VEGF-A165 to VEGFR-2 and increases the efficacy of its signaling. Endothelial cell veins and lymphatic vessels predominantly express NRP-2, with binding sites for VEGF-C and VEGF-D, thus enhancing the signaling through VEGFR-3. More recently, NRP-1 has been involved in the interaction between integrin α5β1 on endothelial cells and the sVEGFR-1 present in the extracellular matrix, leading to angiogenesis [72]. VEGF-A can also directly act on neuron-expressing NRP-1, but the NRP-1-signaling co-receptors have not been identified in this case [68].

NRP-1 contributes to immunity in different ways [73]. NRP-1 has been involved in immune system development and thymocyte differentiation, mainly through binding to SEMA [74,75]. It has also been involved in mediating antigen presentation to T cells by antigen-presenting cells [76]. NRP-1 facilitates cell–cell adhesion through homophilic interactions and colocalizes with CD3 at the contact zone, suggesting a potential role for NRP-1 in the initiation of primary immune responses. However, NRP-1 is expressed by only a limited number of effector T cells and plasmacytoid DCs. Its action in immune cells is believed to be mediated through SEMA/plexin binding and is primarily inhibitory [77]. SEMA3A, secreted by activated DCs and T cells, can bind to NRP-1 on T cells, inhibiting T cell proliferation by blocking actin cytoskeleton rearrangement. Additionally, NRP-1 can exert immune inhibitory effects by binding to TGF-β, a well-known immunosuppressive cytokine also secreted by Tregs [78]. NRP-1 on Tregs can bind to the latent form of TGF-β present in the surrounding tissue fluid or plasma, thereby enhancing immunosuppression.

**Figure 3:**
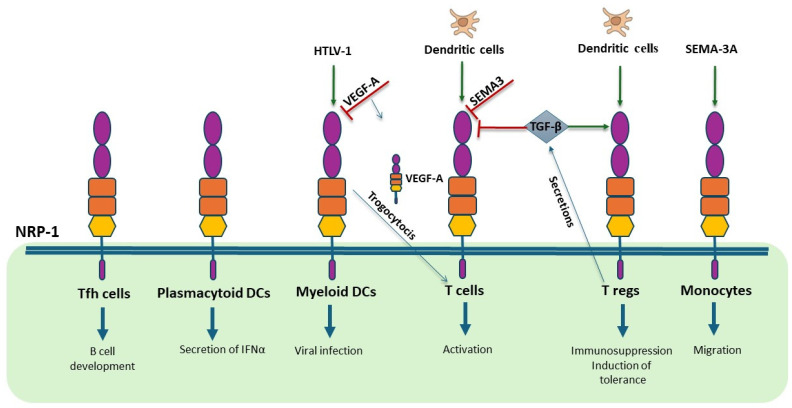
NRP-1 roles in immune cells. NRP-1 is expressed in different cells of the immune system. When expressed in T follicular helper (Tfh) cells, NRP-1 mediates B cell differentiation [81], whereas in plasmacytoid DCs, it induces the secretion of IFNα [82]. Human T cell lymphotropic virus type 1 (HTLV-1) enters myeloid DCs through NRP-1 and VEGF-A can block this infection [84]. Myeloid DCs can transfer NRP-1 and VEGF-A to T cells by trogocytosis [83]. DCs directly activate T cells through NRP-1 but also block T cell activation by secretion of SEMA3 [78]. Regulatory T cells (Tregs) are also directly activated by DCs but also indirectly by secretion of TGF-β that also blocks T cell activation [80]. SEMA-3A/NRP-1 interaction leads to increased migration of monocytes [73]. Parts of the figure are drawn using pictures from Servier Medical Art (https://smart.servier.com).

NRP-1 is expressed by Tregs, but not naive Th cells [79] and plays a key role in promoting long interactions between Treg cells and DCs (Figure 3) [80]. Nrp-1 expression on Tregs gives them an advantage over naive Th cells in the absence of proinflammatory stimuli. Even if VEGFR-1 is present on DCs, neither VEGFR-1 nor VEGFR-2 is expressed on Tregs, excluding the direct involvement of VEGF-A, VEGF-B, and PlGF in immune synapse maintenance.

T follicular helper (Tfh) cells express NRP-1 in secondary lymphoid organs in humans, with its expression being inducible in vitro through interaction with autologous memory B cells (Figure 3). This expression is correlated with plasma B cell precursors, suggesting a role for NRP-1 in B cell differentiation [81].

NRP-1 is expressed on plasmacytoid DCs, and its blockade reduces IFN-α production by these cells, although the precise mechanism remains unclear (Figure 3) [82].

NRP-1 expressed on myeloid DCs can be transferred to T cells by trogocytosis [83]. VEGF-A secreted by human DCs can bind to NRP1 captured by T lymphocytes. Thus, the transfer of NRP-1 to T cells during the immune synapse can transform T lymphocytes into VEGF-A-producing cells. In conjunction with the enhanced VEGFR-2 signaling on endothelial cells facilitated by the NRP-1–VEGF-A complex, the intercellular transfer of NRP-1 may play a role in the remodeling of endothelial vessels in secondary lymphoid organs during inflammation [83].

Myeloid DCs are susceptible to infection by the human T cell lymphotropic virus type 1 (HTLV-1), and NRP-1 has been demonstrated to be essential for viral infection (Figure 3) [84]. Interestingly, VEGF-A acts as a selective competitor of HTLV-1 entry into the cells by binding to the same binding site on NRP-1. In fact, HTLV-1 contains in its envelop protein a structural motif homologous to VEGF-A165.

NRP-1 has been detected on tissue-resident macrophages as well as on tumor-associated macrophages (TAMs), where it plays a critical role in their migration to the tumor hypoxic niche in response to SEMA3A. TAMs express VEGFR-1, and the role of VEGFs in recruiting macrophages has been already underlined. However, no data are available so far to sustain a collaboration of VEGFR-1 and NRP-1 in transducing this chemotactic signal. Bone marrow-derived monocytes expressing NRP-1 were identified as a novel subset by Carrer et al. [85]. These NRP-1-positive monocytes, when injected into tumors, promoted the normalization of tumor vasculature [85]. Similarly, NRP-1 expressed in glioma TAMs has been associated with tumor promotion. Indeed, mice with a TAM-specific deletion of NRP-1 resulted in slower tumor growth, reduced tumor vascularity, and increased survival [86].

Differently from NRP-1, NRP-2 seems to be more broadly expressed in macrophages, during the differentiation from monocytes towards DCs, and in T cell subsets [87]. However, the NRP-2 role in immune cells has not been completely defined.

Altogether, current literature data indicate a role of NRPs in the homeostasis and pathological states of immune cells, but information is still fragmentary, and not all the aspects have been considered, especially the involvement of the VEGFs in activation or repression of immune responses and the possible crosstalk between NRPs and VEGFRs.

## 5. Clinical Advancements and Challenges in Combining VEGF-Targeted Anti-Angiogenic Therapy with Checkpoint Inhibitor Immunotherapy

Due to the amount of data that connects tumor angiogenesis, expression of VEGFs and VEGFRs in the tumor microenvironment, and their role in modulating tumor immune responses, the possibility of combining anti-VEGFs together with anti-checkpoint inhibitors (ICIs) in tumor therapy has been evaluated. Anti-VEGFs exhibit a wide array of effects on the immune system and play a significant role in reversing the tumor immunosuppressive environment [88]. Nevertheless, anti-VEGFs as a single therapeutic approach are likely insufficient to generate a complete and robust immune response against cancer, especially in patients with advanced-stage disease. As a result, anti-VEGFs have been suggested for use in combination with various immunotherapeutic approaches, such as ICIs, to enhance adaptive immune responses. A growing number of clinical trials have been undertaken to evaluate the effectiveness of this combination (Table 1).

High serum levels of VEGF-A in melanoma patients prior to treatment with the anti-CTLA-4 antibody ipilimumab have been associated with reduced overall survival compared to patients with low VEGF-A levels. This finding supports the rationale for targeting VEGF-A in such cases. Consequently, a clinical trial evaluated the combination of ipilimumab and bevacizumab in melanoma patients, demonstrating increased lymphocyte infiltration into tumor tissue [89]. The same combination later showed clinical benefits. Results from the phase I/II clinical trial NCT00790010 demonstrated that the combination of ipilimumab and bevacizumab in patients with metastatic melanoma achieved favorable clinical outcomes, with a disease control rate of 67.4%. The treatment also increased tumor vascular expression of ICAM-1 and VCAM-1, as well as lymphocyte infiltration into the tumors [90].

The most successful results of combination therapies have been reported in renal cell carcinoma (RCC) and hepatocellular carcinoma (HCC). To explore the efficacy of anti-PD-L1 combined with anti-VEGF-A, the phase I study NCT01633970 was proposed and aimed at investigating the safety of atezolizumab plus bevacizumab or chemotherapy in metastatic RCC [91]. A randomized phase II clinical trial (NCT01984242) evaluated the efficacy of atezolizumab plus bevacizumab versus sunitinib as a first-line treatment for RCC. The combination therapy showed significant benefits over sunitinib alone in patients with tumors characterized by high numbers of MDSCs, a robust presence of effector T cells, and elevated PD-L1 expression (≥5%). In contrast, sunitinib was more effective than combination therapy in patients with tumors exhibiting high levels of angiogenesis [14]. A pivotal phase III trial (NCT02420821) is currently underway to confirm the results of this phase II study [92]. Furthermore, a phase III trial comparing avelumab and axitinib combination therapy against sunitinib monotherapy in patients with metastatic RCC in a first-line setting (NCT02684006) revealed that avelumab/axitinib combination significantly prolonged progression-free survival (PFS) compared to sunitinib [93]. Ongoing clinical trials in patients with advanced RCC are showing promising results with combination treatments involving PD-1/PD-L1 inhibitors and anti-angiogenic agents (Table 1).

In patients with advanced HCC, the first randomized phase III clinical trial, IMBRAVE 150 (NCT03434379), demonstrated significant improvements in the co-primary endpoints—PFS and overall survival (OS)—with the combination of atezolizumab and bevacizumab compared to sorafenib. Additionally, the combination therapy delayed the deterioration of quality of life, as reported by patients, and was associated with manageable and tolerable toxicity compared to monotherapy [93]. The safety and efficacy of pembrolizumab combined with lenvatinib were assessed in patients with unresectable HCC during the phase Ib KEYNOTE-524 study. The promising results from this study prompted the design of the phase III LEAP-002 trial (NCT03713593) to evaluate pembrolizumab in combination with lenvatinib as a potential first-line treatment for patients with advanced HCC [94].

In non-squamous non-small cell lung cancer (NSCLC), the phase III IMpower150 clinical trial (NCT02366143) demonstrated that the combination therapy of atezolizumab, bevacizumab, and carboplatin/paclitaxel (ABCP group) significantly improved PFS and OS compared to the control group receiving bevacizumab and carboplatin/paclitaxel [95]. Atezolizumab was approved by the FDA for use in combination with bevacizumab, paclitaxel, and carboplatin as a first-line treatment for patients with metastatic NSCLC following these findings.

Recently, ivonescimab, a tetravalent bispecific mAb simultaneously targeting PD-1 and VEGF-A has been developed, and it is under approval for the treatment of locally advanced or metastatic non-squamous NSCLC, mutated in the EGF receptor, which progressed after therapy with tyrosine kinase inhibitors [96].

In these different combination approaches, the rationale for using a selective VEGF-A inhibitor such as bevacizumab or the tyrosine kinase inhibitors sunitinib or sorafenib, which block different VEGFRs along with other receptors, is not clear. In addition to the clinical aspects, the results of the clinical trials should also be analyzed from a molecular point of view to better understand which anti-angiogenic drug would be more beneficial.

**Table 1:** Clinical trials examining the combination of anti-angiogenic treatments and immune checkpoint inhibitors.

Clinical Trial ID	Study Phase	Agent(s)	Anti-Angiogenic Target	Anti-Tumor Immunity Target	Cancer Types	Refs.
NCT00790010	I	Bevacizumab + Ipilimumab	VEGF-A	CTLA-4	Melanoma	[97]
NCT01950390	II	Bevacizumab + Ipilimumab	VEGF-A	CTLA-4	Melanoma	[97]
NCT01633970	I	Bevacizumab + Atezolizumab	VEGF-A	PD-L1	RCC	[91]
NCT01984242	II	Bevacizumab + Atezolizumab vs. Sunitinib	VEGF-A, VEGFR	PD-L1	RCC	[14,92]
NCT02420821	III	Bevacizumab + Atezolizumab vs. Sunitinib	VEGF-A, VEGFR	PD-L1	RCC	[98,99]
NCT02231749	III	Nivolumab + Ipilimumab vs. Sunitinib	VEGFR, PDGFR	PD-1, CTLA-4	RCC	[100,101]
NCT02493751	I	Axitinib + Avelumab	VEGFR	PD-L1	RCC	[102,103]
NCT02684006	III	Axitinib + Avelumab vs. Sunitinib	VEGFR, PDGFR	PD-L1	RCC	[93,94]
NCT02724878	II	Bevacizumab + Atezolizumab	VEGF-A	PD-L1	RCC	[104]
NCT02853331	III	Pembrolizumab + Axitinib vs. Sunitinib	VEGFR, PDGFR	PD-1	RCC	[98,105]
NCT02811861	III	Pembrolizumab + Lenvatinib vs. Sunitinib	VEGFR, PDGFR	PD-1	RCC	[95]
NCT03721653	II	Bevacizumab + Atezolizumab + FOLFOXIRI	VEGF-A	PD-L1	CRC	[106,107]
NCT03434379	II	Bevacizumab + Atezolizumab	VEGF-A	PD-L1	HCC	[108,109]
NCT03006926	I	Lenvatinib + Pembrolizumab	VEGFR, PDGFR	PD-1	HCC	[110,111]
NCT03713593	III	Lenvatinib + Pembrolizumab vs. Lenvatinib	VEGFR, PDGFR	PD-1	HCC	[112]
NCT02873962	II	Bevacizumab + Nivolumab	VEGF-A	PD-1	OC	[113]
NCT03038100	III	Bevacizumab + Atezolizumab and Chemiotherapy	VEGF-A	PD-L1	OC	[114]
NCT03170960	I	Cabozantinib + Atezolizumab	VEGFR	PD-L1	UC, RCC, NSCLC, HCC	[115,116]
NCT02366143	III	Atezolizumab + Bevacizumab + Paclitaxel/Carboplatin	VEGF-A	PD-L1	NSCLC	[117]
NCT02443324	I	Ramucirumab + Pembrolizumab	VEGFR	PD-1	G/GEJ, NSCLC, UC, BTC	[118]
NCT02572687	I	Ramucirumab + Durvalumab	VEGFR	PD-L1	NSCLC, G/GEJ, HCC	[119]
NCT02856425	I	Nintedanib + Pembrolizumab	PDGFR, VEGFR	PD-1	Advanced solid tumors	[120]
NCT03377023	I/II	Nintedanib + Nivolumab + Ipilimumab	PDGFR, VEGFR	PD-1, CTLA-4	NSCLC	[121]

Abbreviations: CRC, colorectal cancer; G/GEJ, gastric or gastroesophageal junction adenocarcinoma; HCC, hepatocellular carcinoma; NSCLC, non-small cell lung cancer; OC, ovarian cancer; RCC, renal cell cancer; UC, urothelial carcinoma; BTC, biliary tract cancer.

## 6. Innovative Approaches Targeting VEGFs and Tumor-Mediated Immune Suppression

Besides approved clinical trials, additional promising molecules targeting the VEGFs have been developed and are now in the preclinical phase. Among these, mAb D16F7 has been developed against VEGFR-1 that selectively blocks VEGFR-1 dimerization and signal transduction upon binding with VEGFs. This antibody showed efficacy in improving anti-PD-1 responses in melanoma [122]. The D16F7 mAb reduces both in vitro and in vivo chemotaxis of activated M2 macrophages, thereby helping to diminish the immunosuppressive tumor microenvironment.

For VEGF-A ligands, and to a lesser extent for PlGF, there is now a shift towards the development of smaller ligands, such as nanobodies, single-chain monoclonal antibodies, and peptides [123]. This also involves the incorporation of unnatural amino acids and chemical modifications to design and enhance the properties of previously identified molecules, such as improved serum stability and increased affinity [124]. 

Additional strategies such as vaccine combination with anti-angiogenic agents have also been followed. The combination of a vaccine made from peptides of the tumor antigen survivin (SVX vaccine) with sunitinib has been proposed in a colorectal carcinoma model [125]. The therapeutic synergy between the SVX vaccine and sunitinib was highlighted when the vaccine was administered at the end of anti-angiogenic treatment. The initial sunitinib treatment resulted in the promotion of an immune-favorable tumor microenvironment, with pericyte coverage of tumor vessels, rich in NK cells and tumor-infiltrating effector T cells, but poor in MDSCs. This new immune environment may enhance the activity of the vaccine. To investigate whether a positive synergy between lymphangiogenesis and cancer immunotherapy could be leveraged in a cancer vaccine, lethally irradiated tumor cells overexpressing VEGF-C, along with topical adjuvants, were used.

The “VEGF-C vax” induced significant local lymphangiogenesis and enhanced T cell activation in the murine model, both at the intradermal vaccine site and in the draining lymph nodes. This led to higher frequencies of antigen-specific T cells. In fact, the lethally irradiated tumor cells undergo radiation-induced cell death and provide a source of tumor-associated antigens, whereas VEGF-C overexpression activates local lymphatics to proliferate and increase antigen transport to the distal lymph nodes [126].

Preclinical applications of small interfering RNAs (siRNAs) indicate that the next-generation anti-angiogenic compounds could be represented by this class of molecules [127]. However, siRNAs are unstable, and delivery systems should be developed for their usage in clinical applications.

## 7. Conclusions

Different immune cells secrete VEGFs and present the VEGFRs or the co-receptor NRPs. However, the data in the literature are still fragmentary and do not consider the co-expression of different receptors and co-receptors on the same cell type or the presence of different VEGFs in the cell environment, which could lead to opposite results depending on the relative abundance of one VEGF compared to the others. In addition, the investigation of other VEGFs besides VEGF-A should be encouraged for the development of novel anti-angiogenic and anti-inflammatory agents. These new therapeutics may be more useful in the treatment of specific pathologies, alone or in combination with immunotherapies.
